# THE EXPERIENCE OF AGING IN DAILY LIFE IN CROSS-CULTURAL CONTEXTS

**DOI:** 10.1093/geroni/igad104.1533

**Published:** 2023-12-21

**Authors:** Shevaun Neupert, Yuval Palgi

## Abstract

Subjective views of aging capture the many ways that people acknowledge, contemplate, and reflect on aging. These views are related to a wide range of health and well-being indicators including emotional well-being, mental health, chronic health conditions, memory functioning, and even life expectancy. However, most of what we know about subjective views of aging and health is based on W.E.I.R.D. (Western, Educated, Industrialized, Rich, Democratic) samples. The Subjective Aging within Global Everyday ecological Studies (Subjective AGES) consortium was built to address this problem by collecting parallel daily diary data from the United States, Turkey, Germany, United Kingdom, and Israel. Neupert will provide an overview of the consortium and present findings from the U.S. data, focusing on the interaction of daily stressors and uplifts for subjective aging. Can will demonstrate the deleterious effects of daily stressors on subjective aging within the Turkish data. Kornadt will show the beneficial effects of daily uplifts on subjective aging within the German data. Tse will demonstrate that doing personally meaningful and flow-conducive activities predicts feeling younger in the United Kingdom data. Shrira, Palgi, and Neupert will show cultural differences and similarities between Israeli Jews and Arabs and then present cross-country differences comparing the Israeli sample to the American sample. The talk will end by synthesizing the findings from each talk and pointing to future directions for the consortium. Collectively, these findings point to the importance of taking a cultural perspective in the daily relationships between subjective aging and well-being.

